# Nutrient Deprivation Coupled with High Light Exposure for Bioactive Chrysolaminarin Production in the Marine Microalga *Isochrysis zhangjiangensis*

**DOI:** 10.3390/md20060351

**Published:** 2022-05-26

**Authors:** Xiuyuan Ran, Yuhan Shen, Dongjian Jiang, Chenqi Wang, Xinghui Li, Haoyu Zhang, Yunyun Pan, Chenglin Xie, Tonghui Xie, Yongkui Zhang, Changhong Yao

**Affiliations:** 1 Department of Pharmaceutical & Biological Engineering, School of Chemical Engineering, Sichuan University, Chengdu 610065, China; 2020223070113@stu.scu.edu.cn (X.R.); 2020223070122@stu.scu.edu.cn (Y.S.); hyzhang@stu.scu.edu.cn (H.Z.); loadstar@stu.scu.edu.cn (Y.P.); chenglinxie2021@163.com (C.X.); xietonghui@scu.edu.cn (T.X.); zhangyongkui@scu.edu.cn (Y.Z.); 2 Department of Chemical Technology, School of Chemical Engineering, Sichuan University, Chengdu 610065, China; 2019141490229@stu.scu.edu.cn (D.J.); 2019141490227@stu.scu.edu.cn (C.W.); 3 West China School of Pharmacy, Sichuan University, Chengdu 610041, China; 2019141490133@stu.scu.edu.cn

**Keywords:** chrysolaminarin, *Isochrysis zhangjiangensis*, nutrient deprivation, light intensity, antioxidant

## Abstract

Chrysolaminarin, a kind of water-soluble bioactive β-glucan produced by certain microalgae, is a potential candidate for food/pharmaceutical applications. This study identified a marine microalga *Isochrysis zhangjiangensis*, in which chrysolaminarin production was investigated via nutrient (nitrogen, phosphorus, or sulfur) deprivations (-N, -P, or -S conditions) along with an increase in light intensity. A characterization of the antioxidant activities of the chrysolaminarin produced under each condition was also conducted. The results showed that nutrient deprivation caused a significant increase in chrysolaminarin accumulation, though this was accompanied by diminished biomass production and photosynthetic activity. -S was the best strategy to induce chrysolaminarin accumulation. An increase in light intensity from 80 (LL) to 150 (HL) µE·m^−2^·s^−1^ further enhanced chrysolaminarin production. Compared with -N, -S caused more suitable stress and reduced carbon allocation toward neutral lipid production, which enabled a higher chrysolaminarin accumulation capacity. The highest chrysolaminarin content and concentration reached 41.7% of dry weight (%DW) and 632.2 mg/L, respectively, under HL-S, with a corresponding productivity of 155.1 mg/L/day achieved, which exceeds most of the photoautotrophic microalgae previously reported. The chrysolaminarin produced under HL-N (Iz-N) had a relatively competitive hydroxyl radical scavenging activity at low concentrations, while the chrysolaminarin produced under HL-S (Iz-S) exhibited an overall better activity, comparable to the commercial yeast β-glucan, demonstrating *I. zhangjiangensis* as a promising bioactive chrysolaminarin producer from CO_2_.

## 1. Introduction

β-glucan is a kind of polysaccharide that has a variety of biological activities, including immunomodulation [1] as well as anti-tumor [2] and anti-oxidation [3,4] properties, meaning it possesses a tremendous application potential in medicines [5], foods [6], and cosmetics [7]. β-glucan is widely present in the cell walls of fungi (such as mushrooms and yeasts) and plants (including barley, wheat, etc.) [8]. However, among these raw materials, the β-glucan contents are generally relatively low (<10% of dry weight) [9]. Moreover, they also contain other heteropolysaccharides such as chitin [10], which complicates the extraction of β-glucan. Therefore, it is necessary to develop new sources of β-glucan with high productivity and content.

Microalgae are fast-growing single-celled photosynthetic microorganisms. They are considered an ideal source of β-glucan because of their ability to fix carbon dioxide and efficiently accumulate β-glucan under controllable cultivation conditions [11,12]. Microalgal β-glucan is produced mainly in the form chrysolaminarin in Haptophyta and Heterokontophyta, and paramylon in Euglenophyta [11,13]. In particular, chrysolaminarin, a β-glucan-type polysaccharide consisting of a β-1,3-linked backbone with infrequent β-1,6-linked branches [14], is produced by a variety of microalgae such as diatoms (e.g., *Phaeodactylum tricornutum* and *Odontella aurita*) [15], Chrysophytes (e.g., *Poterioochromonas malhamensis* and *Isochrysis galbana*) [16,17], and Eustigmatophytes (e.g., *Nannochloropsis gaditana*) [18]. It is water soluble, stored in the vacuoles, and can account for more than 40% of algal biomass under certain conditions, which makes it easy to extract and purify [15,17]. Chrysolaminarin from microalgae, which is similar to the β-glucan obtained from fungi and plants, though not as extensively researched, has been demonstrated to possess similar anti-oxidation [19,20], immunomodulation [20], and anti-tumor [16] properties, as well as regeneration-promoting activities [17], indicating the potential for this polysaccharide to be applied in food/pharmaceutical fields.

In microalgae, chrysolaminarin is usually accumulated under stress conditions such as nutrient limitations [21,22]. Nitrogen limitation is the most widely used strategy to enhance chrysolaminarin production in microalgae. In the marine diatom *Odontella aurita*, nitrogen limitation has been found to cause large amounts of chrysolaminarin accumulation, with the content reaching 40–60% of sample biomass [21,23]. Phosphorus-limited conditions also induced the accumulation of chrysolaminarin in *Odontella aurita* and *Phaeodactylum tricornutum*, but with much less efficiency than nitrogen limitation [21,24]. The regulation of sulfur concentration in the culture medium for chrysolaminarin production has been shown to be relatively limited, with the only case, to the best our knowledge, showing the effectiveness of sulfur limitation in *Odontella aurita* [21]. More research is needed to demonstrate the effectiveness of this method for chrysolaminarin production in other microalgae. In addition, light intensity is another important factor affecting storage carbohydrate production in microalgae [25]. In general, moderately high light exposure can facilitate chrysolaminarin production [19,26]. However, some exception can occur, since light exposure also influences microalgal lipid accumulation, which can compete with chrysolaminarin production [21]. Therefore, the impacts of light conditions on chrysolaminarin production could be species-dependent, and any potential synergistic effects with nutrient limitation deserve intensive investigation.

The present study identified the marine microalga *Isochrysis zhangjiangensis*, which was previously shown to accumulate carbohydrate as a major carbon sink under nitrogen depletion [27]. The effects of various nutrient deprivations, i.e., nitrogen-, phosphorus-, or sulfur-deprivation, along with an increase in light intensity, on the chrysolaminarin production were investigated in detail. Moreover, the chrysolaminarin produced under nitrogen and sulfur deprivation conditions were extracted and characterized according to their antioxidant activities to investigate their potential applications.

## 2. Results and Discussion

### 2.1. Identification of the Microalga Strain

**Figure 1:**
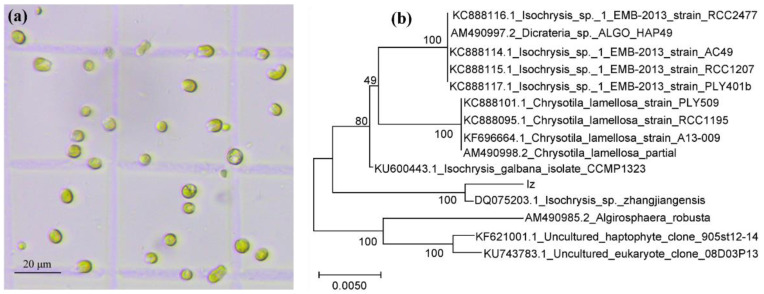
Microscopic image of microalgae in the exponential growth phase (**a**); phylogenetic tree for isolated strain (Iz) constructed by neighbor-joining algorithm based on 18S rDNA sequences through MEGA-X software (https://www.megasoftware.net/, accessed on 24 March 2022) with 1000 bootstraps (**b**).

The microalga was initially isolated from the South China Sea near Zhanjiang, China, and was separated and purified by the plate streak method. Microscopic observation showed that the microalga cells had a golden or brown color, and they were spherical or oval with a diameter of 5 to 7 μm (Figure 1a). The findings of 18S rDNA sequence phylogenetic analysis showed that it possesses 99.65% nucleotide similarity to the strain *Isochrysis zhangjiangensis* (Accession number DQ075203.1) with a bootstraps value of 100% (Figure 1b). Combined with the morphological analysis and molecular identification results, the alga strain was identified as *Isochrysis zhangjiangensis*.

### 2.2. Chrysolaminarin Production under Different Nutrient Deprivations and Light Intensities

#### 2.2.1. Biomass Production

**Figure 2:**
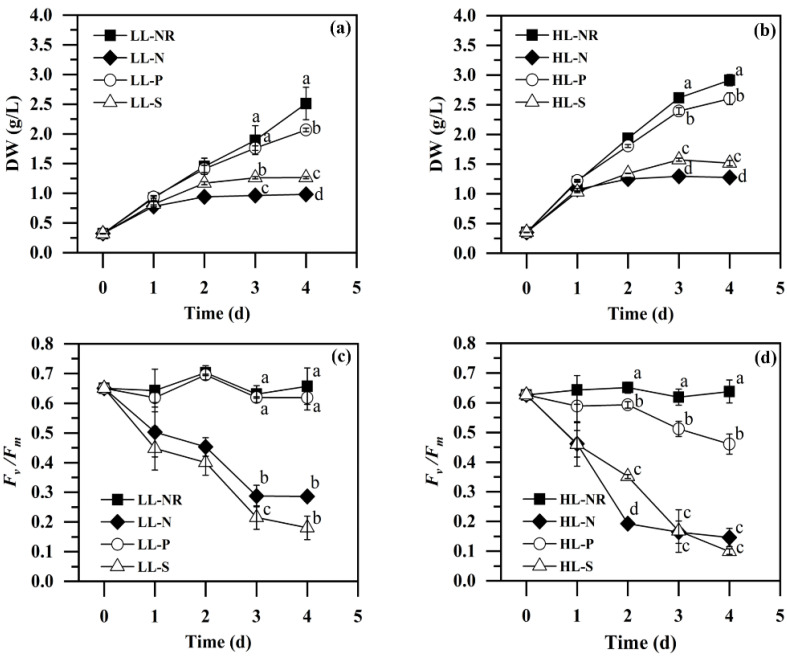
Biomass production (**a**,**b**) and photosynthetic activity (**c**,**d**) of *Isochrysis zhangjiangensis* cultivated with different nutrient deprivations under low light (LL, **a**,**c**) and high light (HL, **b**,**d**) intensities. Values are expressed as mean ± standard deviation of three biological replicates. Values with different letters in the same cultivation day represent significant differences (*p* < 0.05) between various cultivation conditions.

Macronutrients (such as N, P, and S) are essential for microalgae growth. Under macronutrient deprivation, microalgae cells can grow at a reduced rate for several days via the recycling of nutrients stored in the cells from nutrient-rich compounds such as protein for N and S and polyphosphate for P [27]. As shown in Figure 2a,b, biomass could accumulate under all the nutrient-deprivation conditions tested, but the biomass productions declined compared with the NR culture under both LL and HL, especially for -N and -S. -P caused less decline in biomass production relative to -N and -S, with final biomass concentrations of 2.07 g/L and 2.60 g/L on Day 4 under LL and HL, respectively, which were 64–128% higher than those under -N or -S. Similar phenomenon have been observed with other microalgae such as *Dunaliella salina* and *Coccomyxa* sp., in which P deficiency led to a much milder impact on cell growth compared with N or S starvation [28,29]. P is usually stored in microalgae as polyphosphate, which can be released to supply P when cells are exposed to extracellular P deficiency [30]. In addition, the phospholipids in the membrane tend to be replaced by non-phosphorus lipids when microalgae acclimate to P starvation [24]. This can explain why biomass production under P deprivation was not severely affected. It should be noted that -S showed higher biomass production than -N throughout the cultivation period under both LL and HL (Figure 2a,b). The final biomass concentration in the S-deprived cultures reached 1.3 g/L under LL and 1.5 g/L under HL, representing 30% and 15% improvements, respectively, compared with N-deprived cultures. In *Chlamydomonas reinhardtii*, increased growth rates amounting to a 12% higher biomass production were observed under S deprivation compared with that under N deprivation [31], which coincided with the results with *I. zhangjiangensis* in the present study. It is assumed that S is 10 times less abundant in microalgae than N, and hence the demand for S is relatively easier to meet through the recycling of intracellular stores, which partially facilitates growth under S deprivation relative to N deprivation [32]. These findings demonstrate that S deprivation could be more favorable than N deprivation for biomass accumulation.

Light is an important factor affecting the growth of microalgae cells. It can be observed from Figure 2a,b that biomass accumulation was faster under HL than that under LL in all of the conditions tested. The final biomass concentration under HL reached 2.9 g/L, 1.3 g/L, 2.6 g/L, and 1.5 g/L under NR, -N, -P, and -S, respectively, representing 16.0%, 29.6%, 25.9%, and 19.9% improvements, respectively, compared with their corresponding counterparts under LL. These results comply with the general rule that higher light exposure leads to more biomass accumulation in photoautotrophic microalgae because light is the sole energy source for cell growth and carbon fixation [33]. As a result, the biomass productivities on Day 4 under HL were 17.1%~29.8% higher than those under LL, reaching 640.73 mg/L/day, 230.67 mg/L/day, 560.52 mg/L/day, and 290.9 mg/L/day under NR, -N, -P, and -S, respectively (Table 1).

#### 2.2.2. Photosynthetic Activity

The chlorophyll fluorescence parameter *F_v_/F_m_*, the potential maximum quantum efficiency of PS II, is regarded as an internal metric for studying the relationship between photosynthesis and the environment. It is an intrinsic and sensitive indicator of environmental stress conditions, which manifest as a decrease in *F_v_/F_m_* [34]. As shown in Figure 2c, under LL, -N and -S caused a significant (*p* < 0.05) and dramatic decline in *F_v_/F_m_* throughout cultivation, with 44% (from 0.650 to 0.286) and 28% (from 0.650 to 0.180), respectively, remaining on Day 4. In contrast, -P resulted in marginal changes compared with NR, with the *F_v_/F_m_* maintained at high levels (above 0.62) during the entire cultivation period, although an insignificantly (*p* > 0.05) lower *F_v_/F_m_* value could be detected on Day 4. As discussed above, many microalgae can store polyphosphate intracellularly, which can be recycled to temporarily provide phosphorus for cells to acclimate to P deficiency, thus making photosynthesis and biomass production less affected over a short period [30]. However, studies of microalgae subjected to -N or -S conditions have reported that the proteins associated with the photosynthetic apparatus (especially D1 protein of PS II) and the Calvin–Benson–Bassham Cycle (CBBC) (especially ribulose-1,5-bisphosphate carboxylase/oxygenase) declined within a short time, leading to diminished photosynthetic capacity [35,36,37,38]. In the present study, the protein content of cells in -N and -S was only 53% of the level in NR, while it remained 85% in -P (Table 1). Collectively, this demonstrates that -N and -S led to severe stress and drastically reduced photosynthetic activity in *I. zhangjiangensis*, whereas -P exerted much less stress with almost unaffected photosynthesis, which is consistent with the result of the biomass production analysis, i.e., that *I. zhangjiangensis* accumulated 62–110% more biomass under -P than under -N or -S (Figure 2a).

High light exposure generally followed the same trend as under LL, but it accelerated the decline in *F_v_/F_m_* in all the cultures with nutrient deprivations compared with low light conditions, with 23% (from 0.626 to 0.146), 74% (from 0.626 to 0.461), and 16% (from 0.626 to 0.098) remaining under -N, -P, and -S, respectively, on Day 4 (Figure 2d). High light exposure can cause excessive electron transfer through the photosynthetic apparatus in microalgae, leading to oxidative stress and photoinhibition, and the aggravation of photosynthetic activity can be further aggravated when combined with nutrient deprivation conditions [33]. Specifically, the decline in photosynthetic activity was significant (*p* < 0.05) under -P and HL, which indicates the presence of an obvious stress therein, although -N and -S suffered from much stronger stress compared with -P, as was the case under LL. It is worth noting that -S caused an overall lower *F_v_/F_m_* value compared with -N under LL. Conversely, however, -N led to a more rapid decline in *F_v_/F_m_* compared with -S under HL, especially during the first two days, although a slightly lower *F_v_/F_m_* value was still observed in -S on the final day (Day 4) (Figure 2c,d). This suggests that -S could generally cause more severe stress than -N, but the regulation of cell metabolism in *I. zhangjiangensis*, as revealed by photosynthetic activity, was more sensitive to high light exposure in -N than in -S. In addition, it appears that the PSII activity does not necessarily precisely correspond to biomass accumulation, since -S resulted in more biomass production than -N under both LL and HL, which was against the trend of *F_v_/F_m_* herein (Figure 2a–d). This finding could be ascribed to the production of storage compounds such as carbohydrate in microalgal cells under nutrient deprivations, as shown in the following sections.

#### 2.2.3. Chrysolaminarin Production

**Figure 3:**
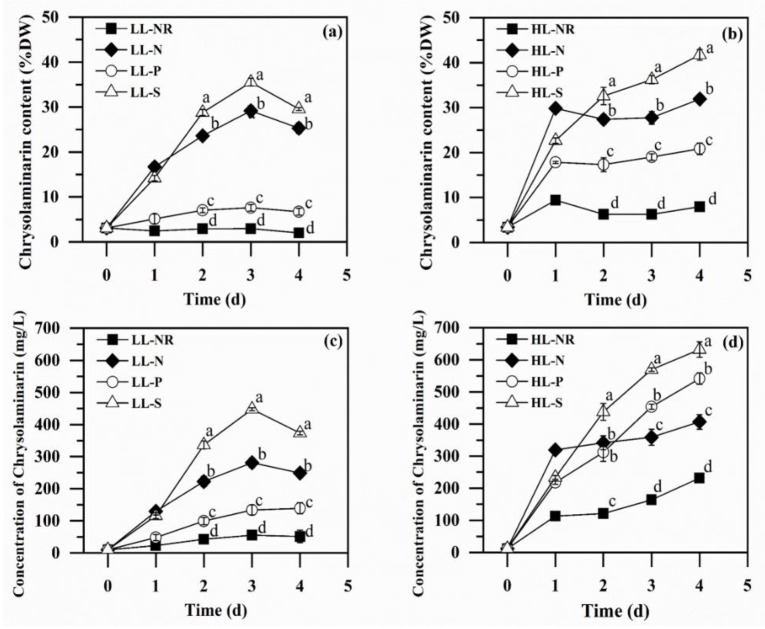
Chrysolaminarin content (**a**,**b**) and chrysolaminarin concentration (**c**,**d**) of *Isochrysis zhangjiangensis* cultivated with different nutrient deprivations under low light (LL, **a**,**c**) and high light (HL, **b**,**d**) intensities. Values with different letters in the same cultivation day represent significant differences (*p* < 0.05) between various cultivation conditions.

Chrysolaminarin is the main storage carbohydrate in the genus *Isochrysis* under stress conditions [39]. As shown in Figure 3a,b, chrysolaminarin accumulated in all the nutrient deprivation conditions tested under both LL and HL, with -S being the most efficient inducer, followed by -N and -P. Cell morphology analysis showed that -N and -S led to much larger cells than NR and –P, with –S having the largest cells with a round shape, suggesting that large amounts of carbohydrate were accumulated intracellularly in -S and -N (Figure S1), a finding in line with previous studies [31,39]. In all cultures under LL, the chrysolaminarin content increased continuously under nutrient deprivation until a peak value occurred on Day 3, with the maximum chrysolaminarin content reaching 29.2%DW, 7.6%DW, and 35.5%DW in -N, -P, and -S cultures, respectively, representing 8.7-, 1.5-, and 10.8-fold enhancements compared with the NR culture (Figure 3a). Similarly, the chrysolaminarin concentration closely mirrored the variation profile of the chrysolaminarin content, and peak values of 280.9 mg/L, 133.9 mg/L, and 447.7 mg/L were observed in -N, -P, and -S cultures, respectively, on Day 3, levels 1.4 to 7.1 times higher than that in the NR culture (Figure 3c). High light exposure further promoted chrysolaminarin content and chrysolaminarin concentration in all the cultures. As shown in Figure 3b,d, a more rapid increase in the chrysolaminarin content and concentration was observed under HL compared with the corresponding cultures under LL, especially on the first day. Under HL, the highest chrysolaminarin content (31.9%DW for -N, 20.8%DW for -P, and 41.7%DW for -S) and concentration (406.6 mg/L for -N, 541.3 mg/L for -P, and 632.1 m g/L for -S) were both obtained on Day 4, representing 9.2–17.4%, 173.6–304.2%, and 17.4–41.1% improvements for -N, -P and -S cultures, respectively, compared with the corresponding maximum levels achieved under LL on Day 3 (Figure 3). Even under NR, in which the environment was not ideal for carbohydrate accumulation, enhanced chrysolaminarin production could also be observed under HL relative to LL (8.0%DW and 232.1 mg/L vs. 2.0%DW and 51.5 mg/L). Similarly, Christian Schulze et al. [11] found that after the light intensity increased from 50 µE·m^−2^·s^−1^ to 150 µE·m^−2^·s^−1^, the content of β-glucan in *Scenedesmus obtusiusculus* A189 cells increased from 6.4% to 19.5%. Chrysolaminarin production was also improved in a marine diatom, *Odontella aurita*, when light intensity increased from 100 to 300 µE·m^−2^·s^−1^ [19], which likewise coincides with the results for *I. zhangjiangensis* herein.

Microalgal storage carbohydrates are usually accumulated under stress conditions such as nutrient starvation and/or high light exposure [40]. This is regarded as a general response of many microalgae that enables them to acclimate to the unfavorable environments they are subjected to because carbohydrate can serve as a sink for excessive carbon and electrons when protein and polar lipid synthesis is diminished under stress conditions [41,42,43]. Therefore, stress is considered indispensable for storage carbohydrate accumulation in microalgae. Herein, it is demonstrated that the chrysolaminarin production ability of *I. zhangjiangensis* (as reflected by its content) under -N and -S conditions was much greater than under the -P one, which could be ascribed to the stronger stress under -N and -S relative to -P, as indicated by the lower *F_v_/F_m_* value and greatly reduced biomass production (Figure 2). As in the case of *I. zhangjiangensis* reported herein, in the marine microalga *Odontella aurita*, -N and -S have also been demonstrated to be superior to -P for chrysolaminarin accumulation [21]. The improvement of chrysolaminarin production under HL could also be attributed to the enhanced stress compared with that under LL, as demonstrated by the reduced *F_v_/F_m_* value (Figure 2c,d). The promotion of chrysolaminarin production with the increased light intensity was enormous in the -P cultures, with the chrysolaminarin content enhanced from 7.6%DW under LL to 20.8%DW under HL (Figure 3a,b), consistent with the significant decrease in *F_v_/F_m_* from 0.619 under LL to 0.461 under HL (Figure 2c,d). Because of the relatively high photosynthetic activity and the consequent high biomass production capacity, the chrysolaminarin concentration in -P under HL reached 541.4 mg/L, with a chrysolaminarin productivity of 132.4 mg/L/day, which surpassed the levels in -N by ~34%, though it was still inferior to that in -S by ~15% (Figure 2d, Table 1).

The present study demonstrates that -S, rather than -N, is the best strategy for chrysolaminarin production in *I. zhangjiangensis* under both LL and HL. In green algae, such as *Tetraselmis subcordiformis*, *Chlorella vulgaris* Beijerinck CCALA924, and *Chlamydomonas reinhardtii* CC-124 and CC-125, -S has been shown to be superior to -N for storage carbohydrate (starch) production, which is consistent with the present study [32,44,45]. However, in the marine diatom *Odontella aurita*, -N displayed higher carbohydrate and β-1,3-glucan accumulation than in -S, which is different from the situation described herein [21]. The better chrysolaminarin accumulation ability of *I. zhangjiangensis* in -S relative to -N could be ascribed to the following reasons. Firstly, -S maintained more suitable stress than -N for chrysolaminarin production. As discussed above, stress leads to storage carbohydrate production in microalgae, and its accumulation is usually positively associated with the stress level. However, since carbohydrate biosynthesis requires photosynthesis, an adequate photosynthetic activity with moderate stress is also required [45,46]. Under LL, -S exerted more stress on the microalgae than -N, as reflected by the overall lower *F_v_/F_m_* value (Figure 2c), possibly resulting in higher chrysolaminarin accumulation (Figure 3a,c, Table 1). However, under HL, the higher chrysolaminarin accumulation in -S could be partially due to the maintained photosynthetic activity. It was clear that -N led to a drastic reduction in *F_v_/F_m_* during the first two days, with a low photosynthetic activity remaining (*F_v_/F_m_* < 0.19), while in -S the *F_v_/F_m_* value remained above 0.35 (Figure 2d). Therefore, the chrysolaminarin accumulation halted in -N, whereas it continued in -S, resulting in a higher chrysolaminarin content, concentration, and productivity (Figure 3b,d, Table 1). In addition, from the perspective of carbon partitioning, the neutral lipid synthesis in -S seemed less active than in -N, which could enable more carbon to be directed into chrysolaminarin synthesis. As shown in Table 1, the protein content showed no significant difference (*p* < 0.05) between -N and -S, while the neutral lipid levels in -N were significantly higher (*p* < 0.05) than those in -S, with 69% and 116% more neutral lipid obtained under LL and HL, respectively. This indicates that neutral lipid synthesis, which is considered to be a competitive process for carbon allocation to carbohydrate under nutrient starvation [40,47], was less favorable in -S than in -N, which could in turn facilitate chrysolaminarin synthesis. In the green microalga *Parachlorella kessleri*, sulfur deprivation also led to less accumulation of neutral lipid than nitrogen deprivation [48], similar to the case in *I. zhangjiangensis* described herein. However, in *Chlamydomonas reinhardtii*, sulfur deprivation was reported to stimulate more lipid and carbohydrate accumulation than was achieved under nitrogen deprivation [31,32]. The different carbon allocation profile of *I. zhangjiangensis* in response to nitrogen and sulfur starvation requires further investigation. Another advantage of using -S over -N for chrysolaminarin production in *I. zhangjiangensis* is the higher biomass production, as shown in Figure 1 and Table 1.

In summary, the present study demonstrated that -S coupled with HL was the most favorable strategy to induce chrysolaminarin production in *I. zhangjiangensis*. The maximum chrysolaminarin content and concentration reached 41.7%DW and 632.2 mg/L respectively in -S on Day 4, and a chrysolaminarin productivity of 155.1 mg/L/day and biomass productivity of 291.5 mg/L/day were obtained (Figure 2b,d, Table 1). Compared with the algal strains in terms of chrysolaminarin production ability under photoautotrophic conditions reported in the literature (Table 2), *I. zhangjiangensis* was superior to most of the strains, such as *Tribonema utriculosum*, *Rhodosorus* sp. SCSIO-45730, and *Phaeodactylum tricornutum*, although it was inferior to *Odontella aurita* under optimal conditions. Nevertheless, *I. zhangjiangensis* is a promising candidate for photosynthetic chrysolaminarin production from CO_2_.

### 2.3. Preliminary Characterization of Chrysolaminarin from I. zhangjiangensis

**Figure 4:**
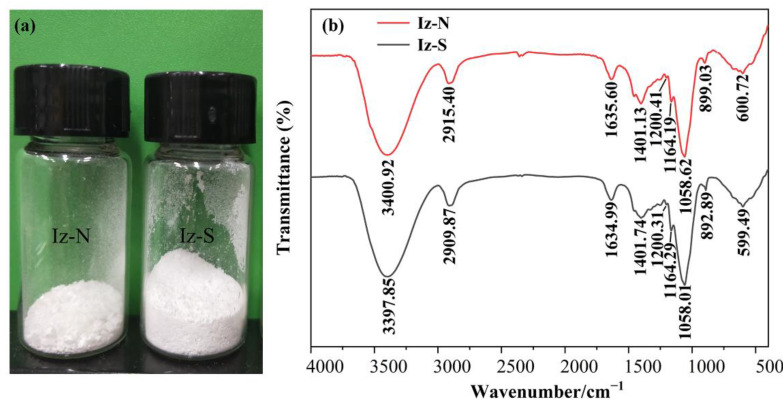
The picture (**a**) and the FTIR spectra (**b**) of polysaccharides from *I. zhangjiangensis* cultivated under nitrogen or sulfur deprivation coupled with high light exposure. The polysaccharides were named Iz-N and Iz-S, respectively.

-N and -S were demonstrated to induce a high level of chrysolaminarin accumulation, with a chrysolaminarin content of 32%DW~42%DW obtained under HL (Figure 2). Therefore, the chrysolaminarin was extracted from the algae cultivated in -N and -S under HL (named Iz-N and Iz-S, respectively, Figure 4a) for preliminary characterization of its structure and activity.

#### 2.3.1. Chemical Composition

Chrysolaminarin is a kind of water-soluble polysaccharide [17]. After hot-water extraction followed by trichloroacetic acid treatment for protein elimination and ethanol precipitation, the carbohydrate fraction accounted for ~90% of the extracted polysaccharides in both Iz-N and Iz-S, with protein and lipid contents of less than 1.5%. The chrysolaminarin fraction in Iz-N and Iz-S was enzymatically determined (K-YBGL 02/21, Megazyme, Bray, Ireland) to be 86.55% and 87.07%, respectively, indicating that the chrysolaminarin accounted for more than 95% of the total carbohydrate, reflecting a high purity (Table 3).

#### 2.3.2. FTIR Spectra Analysis

Figure 4b shows the FTIR spectra of the polysaccharides. In general, Iz-N and Iz-S had very similar characteristic FTIR spectra. The strong absorbance band at 3400.92 cm^−1^ could be attributed to the stretching vibration of O-H groups. The stretching vibration of the C-H bond produced a characteristic absorption band at 2915.40 cm^−1^ [51]. The absorbance band at 1635.60 cm^−1^ corresponds to the asymmetric stretching vibration of C=O in -CHO. The asymmetric vibrations of the glycosidic bond C-O-C appeared near 1164, 1200, and 1058 cm^−1^, indicating the presence of the pyranyl saccharide ring in the sample [52]. The above characteristic bands indicated that the samples were mainly composed of carbohydrates, which is consistent with the results from the chemical analysis (Table 3). The weak absorbance at 899.03 cm^−1^ represented the anomeric carbon stretching vibration of β-type hetero polysaccharide, which is a typical glycosidic bond configuration for chrysolaminarin [53]. In addition, the FTIR spectra of Iz-N and Iz-S resembled that of the chrysolaminarin from *Odontella aurita* [19]. Collectively, combined with the FTIR analysis (Figure 4) and enzymatic quantification results (Table 3), Iz-N and Iz-S could be recognized as chrysolaminarin.

#### 2.3.3. Antioxidant Activity

β-glucans have been previously reported to have antioxidant activities [54]. Chrysolaminarin from microalgae such as the marine diatoms *Odontella aurita* and *Phaeodactylum tricornutum* had been shown to have DPPH radical scavenging activity and hydroxyl radical scavenging activity [19,20]. In the present study, the antioxidant activities of Iz-N and Iz-S were characterized. For comparison, β-glucan from yeast (Y-BG, purchased from Shanghai Yuanye Bio-Technology Co.,Ltd., Shanghai, China), which is widely used in the food/pharmaceutical industries and aquaculture was also analyzed.

##### Hydroxyl Radical Scavenging Activity

**Figure 5:**
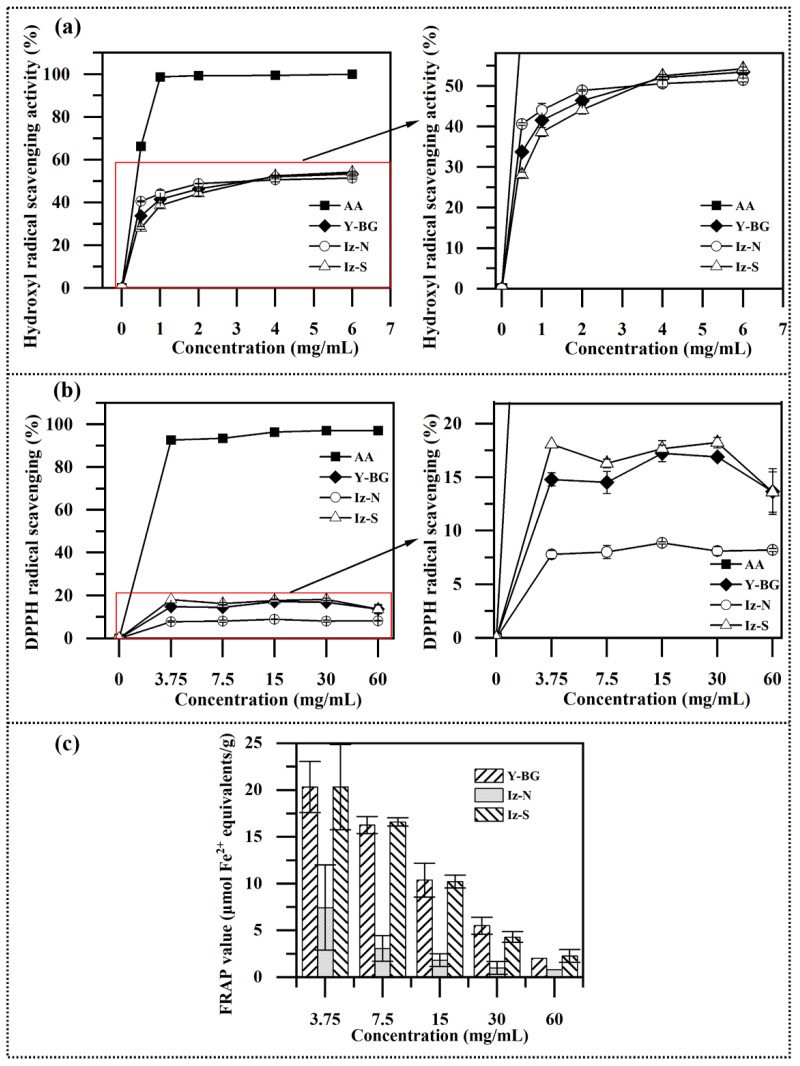
The antioxidant activity of the chrysolaminarin extracted from *I. zhangjiangensis* cultivated under nitrogen or sulfur deprivation coupled with high light exposure (Iz-N and Iz-S, respectively). β-glucan from yeast (Y-BG) was used for comparison. Ascorbic acid (AA) was used as a positive control. (**a**) Scavenging of hydroxyl radicals; (**b**) Scavenging of DPPH radicals; (**c**) Ferric reducing antioxidant power (FRAP) activity. Values are expressed as mean ± standard deviation of two technical replicates.

The hydroxyl radical is considered a harmful free radical because it can cause severe damage to neighboring biomolecules. As shown in Figure 5a, the three polysaccharides exhibited a relatively strong scavenging effect on hydroxyl radicals, and this scavenging effect was enhanced by the increase in the polysaccharide concentration. The maximum scavenging rate reached ~53% at the concentration of 6 mg/mL in all the three polysaccharides, with very little difference between them. However, the scavenging capacity was much lower than that of ascorbic acid, which reached a plateau of 98.7% at 1 mg/mL. The chrysolaminarin CL2 isolated from marine diatom *O. aurita* at a concentration of 10 mg/mL has been shown to have a scavenging rate of 83.54% for hydroxyl radicals [19], indicating better scavenging activity than for the chrysolaminarin reported herein, and the authors also reported that the hydroxyl radical scavenging activity varied depending on the source. The scavenging rate of β-glucan extracted from black yeast or oats with a concentration of 10 mg/mL (1.0 *w/v*%) is reported to be less than 10%, while that extracted from barley can reach about 60% [4]. Overall, the chrysolaminarin extracted from *I. zhangjiangensis* cultivated under different nutrient deprivations (Iz-N and Iz-S) showed almost identical maximum hydroxyl radical scavenging activity, which was moderate and comparable to Y-BG. It should be noted that the scavenging rate of Iz-N and Iz-S at 1 mg/mL reached 44.0% and 38.6%, respectively (Figure 5a), a better performance than for the chrysolaminarin CL2 from *O. aurita*, which had a scavenging rate of less than 20% at this concentration [19]. In particular, at the low concentration of 0.5 mg/mL, the scavenging rate of Iz-N reached 40.6%, which was 45% and 20% higher than for Iz-S and Y-BG, respectively (Figure 5a). This scavenging rate also exceeded many β-glucan derivatives from yeast (scavenging rate of less than 28%) and oat β-glucan (scavenging rate of 5%) at similar polysaccharide concentrations (0.5~0.8 mg/mL) [3,55]. Taken together, these findings indicate that the chrysolaminarin extracted from *I. zhangjiangensis* has a relatively competitive hydroxyl radical scavenging activity under low concentrations, especially in the case of Iz-N. This feature would be favorable for the economic and safe application of the polysaccharide as an antioxidant.

##### 1,1-Diphenyl-2-picrylhydrazyl (DPPH) Radical Scavenging Activity

DPPH is a stable free radical that can accept an electron or hydrogen radical to become a stable diamagnetic molecule, making it a useful tool to evaluate antioxidant activity [56]. As shown in Figure 5b, the DPPH radical scavenging capacity of all three polysaccharides reached a maximum level at the concentration of 3.75 mg/mL, with the scavenging rate following the order Iz-S > Y-BG > Iz-N. The highest DPPH radical scavenging rate observed for Iz-S was 18%, which was weak relative to other β-glucans such as the chrysolaminarin from diatom *Phaeodactylum tricornutum* and β-glucans from oat or brewers’ yeast, which have been found to exhibit optimum DPPH radical scavenging rates of more than 50% [3,20]. Nevertheless, the DPPH radical scavenging rate of Iz-S was 1.5- to 2-fold greater than that of chrysolaminarin CL2 from *O. aurita* at concentrations of 5~10 mg/mL, although the scavenging rate of CL2 has been shown to reach 42% at high concentration (100 mg/mL) [19], whereas for Iz-S no further enhancement of activity was observed with the increase of polysaccharide concentration (Figure 5b).

##### Ferric Reducing Antioxidant Power (FRAP) Activity

A FRAP assay treats the antioxidants as reductants in a redox linked colorimetric reaction, with the value reflecting the reducing power of antioxidants, which is used for a rapid measurement of the total antioxidant capacity of the sample [57]. As shown in Figure 5c, Iz-S had comparable FRAP values to Y-BG at all of the concentrations tested, but these values were 1.7- to 4.5-fold greater than those of Iz-N. At a concentration of 3.75 mg/mL, the FRAP value of Iz-S reached 20.3 μmol Fe^2+^ equivalents/g, which is superior to many commercially available glucans such as carboxymethyl yeast β-glucan C90, oat glucan (SymGlucan), and *Schizophyllum commune* glucan, yet inferior to oat β-glucans [57].

The present study demonstrated that the chrysolaminarin produced from *I. zhangjiangensis* has certain antioxidant activities, but these varied depending on the conditions under which the algae were grown. In general, Iz-N possessed better hydroxyl radical scavenging activity at low concentrations, while Iz-S had higher DPPH radical scavenging capacity and FRAP activity. Different cultivation conditions could affect the enzymes related to the biosynthesis of the polysaccharides, generating polysaccharides with varied structures and hence exhibited different activities. It has been recently reported that the exopolysaccharide extracted from the green alga *Botryococcus braunii* exhibited a changed structure and enhanced antioxidant activity when the alga had been exposed to a high cobalt environment [58]. A detailed comparative characterization of the structures of Iz-N and Iz-S would be of interest to dissect the structure–activity relationship. In particular, the chrysolaminarin Iz-S produced from *I. zhangjiangensis* under HL-S showed an overall comparable or even higher antioxidant activity relative to yeast β-glucan (Y-BG), which exemplifies the potential for this polysaccharide to be applied as an antioxidant in the food/pharmaceutical industries or aquaculture.

## 3. Materials and Methods

### 3.1. Algal Strain and Culture Conditions

The microalga was initially isolated from the South China Sea near Zhanjiang, China in 2013 by Dr. Liangping Ni, Shanghai Guangyu Biological Technology Co., Ltd., Shanghai, China. The microalga was purified with the plate streak method by our research group. It was cultivated in artificial seawater (ASW: NaCl 21.22 g/L, NaHCO_3_ 0.174 g/L, MgCl_2_·6H_2_O 9.034 g/L, CaCl_2_ 1.033 g/L, Na_2_SO_4_ 3.407 g/L, KCl 0.357 g/L, KBr 0.0862 g/L, H_3_BO_3_ 0.023 g/L) enriched with nutrients based on a modified f/2 medium of the following composition: NaNO_3_ 600 mg/L, NaH_2_PO_4_ 20 mg/L, Na_2_SiO_3_ 30 mg/L, FeCl_3_·6H_2_O 3.15 mg/L, EDTA·2Na 4.36 mg/L, CuCl_2_ 8.2 µg/L, Na_2_MoO_4_ 6.3 µg/L, ZnCl_2_ 21 µg/L, CoCl_2_·6H_2_O 0.01 mg/L, MnCl_2_·4H_2_O 0.18 mg/L, vitamin B_12_ 0.001 mg/L, vitamin B_1_ 0.2 mg/L, biotin 0.001 mg/L. The medium was sterilized before use by autoclaving at 115 °C for 30 min. The algal cells were grown in a shaking flask step by step under continuous light (60 µE·m^−2^·s^−1^) at 25 °C, shaking manually more than three times a day.

### 3.2. Morphology Identification and 18S rDNA Gene Sequence Analysis

The morphological characteristics of microalgae were observed by an optical biological microscope (BMC513-IPL, Phenix Optics Co., Ltd., Shenzhen, China). The extraction of algae genomic DNA adopted a modified CTAB method [59]. The main steps were as follows: Algae cells were lysed with 600 µL CTAB buffer at 65 °C for 1 h using a water bath and cooled to room temperature. An equal volume of PCI extraction solution (phenol: chloroform: isoamyl alcohol 25:24:1 (*v/v*)) was then added, and the resultant mixture was mixed well and refrigerated for 5 min. The upper aqueous phase was collected by centrifugation at 4 °C, 12,500 rpm for 10 min. The supernatant was extracted again by adding an equal volume of PCI. Pre-cooled isopropanol with 0.8 times of the volume was added to the aqueous solution recovered from the previous step and placed at 4 °C for half an hour. Then, it was centrifuged with 12,000 rpm at 4 °C for 20 min, discarding the supernatant and washing twice with cold 70% ethanol. After evaporating the residual ethanol, 20 µL TE buffer was added to dissolve the precipitate and stored it at −20 °C.

Eukaryotic 18S rDNA universal primers (forward, 5′-CCAACCTGGTTGATCC TGCCAGTA-3′; reverse, 5′-CCTTGTTAACG- ACTTCACCTTCCTCT-3′) [60] synthesized by Sangon Biotech Co., Ltd. (Shanghai, China) were used to amplify the18S rRNA gene. The PCR program for amplification was 95 °C for 5 min, 30 cycles of 94 °C for 45 s, 55 °C for 45 s, and 72 °C for 1 min, and then at 72 °C for the last 10 min of extension. Sangon Biotech Co., Ltd. (Shanghai, China) purified and sequenced the PCR products. The resulting 18S rRNA gene sequences were aligned and compared with those in the GenBank database of the National Center for Biotechnology Information (NCBI) by a Basic Local Alignment Search Tool (BLAST) search. Using MEGA-X software with the multiple alignment program CLUSTAL W, a neighbor-joining tree with 1000 bootstraps was constructed.

### 3.3. Experimental Design

In the logarithmic growth phase, the microalgae were collected by centrifugation and then washed twice with sulfate-free ASW. ASW enriched with nutrients based on a modified f/2 medium was used as the basic medium for cultivation, and the composition of culture medium with different nutrient conditions was shown in Table S1. After being inoculated into different nutrient conditions at the same initial cell density of 0.3 g/L, the microalgae were cultured in a cylindrical glass column (height 450 mm, diameter 45 mm) [61] with a 500 mL working volume at different light intensities. Two different light intensities, designated as high light (150 µE·m^−2^·s^−1^) and low light (80 µE·m^−2^·s^−1^), were used to evaluate the influence of light intensity on cell growth and chrysolaminarin accumulation. The cultures were aerated by bubbling with a 2% CO_2_-enriched air at a flow rate of 0.25 vvm in an environment maintained at 25 °C. Three independent biological replicates were set for each experimental condition.

### 3.4. Experimental Design

#### 3.4.1. Growth Measurement

The cell growth was estimated via the optical density of the culture, which was measured at 750 nm (OD_750_) on a spectrophotometer (AOE, UV/Vis A-360, Shanghai, China). Biomass production was evaluated with a linear regression standard curve between OD_750_ and dry cell weight as follows: DW = 0.9675 × (OD_750_) − 0.3046 (R^2^ = 0.996), where DW is the dry cell weight (DW, g/L) measured with a gravimetric method according to [62].

The number of cells was counted with a hemocytometer. A linear regression standard curve between optical density OD_750_ and cell concentration was obtained as follows: Y(×10^7^ cells/mL) = 0.9806 × (OD_750_) − 0.5982 (R^2^ = 0.993).

#### 3.4.2. Chrysolaminarin Content

According to Granum and Myklestad [63], chrysolaminarin was extracted with sulfuric acid (0.05 mol/L, 5 mL) at 60 °C for 10 min. The extracts were collected by centrifugation at 10,000× *g* for 5 min. The anthrone-sulfuric acid method [64] was used to quantify chrysolaminarin in the extract with a minor modification. An aliquot of 2.5 mL of anthrone-sulfuric acid solution (containing 0.1 g of anthrone, 87 mL of concentrated sulfuric acid, 30 mL of deionized water) was added to 0.5 mL of the above extracts. After heating in boiling water for 10 min, the reaction mixture was rapidly cooled to room temperature, and the absorbance was measured at 621 nm wavelength. The content of chrysolaminarin was determined by comparison with a calibration curve made with glucose with a calibration factor of 0.9 (glucose to polyglucan).

#### 3.4.3. Protein Content

The protein extraction process was carried out according to the ‘standard method’ described by Rausch [65] with minor modifications. The protein was extracted with 0.5 mol/L sodium hydroxide solution in a water bath at 80 °C for 10 min, and the supernatant was collected by centrifugation at 10,000× *g* for 3 min. BCA protein detection kit (Beyotime Biotechnology, Shanghai, China) was used to determine the protein concentration of the extracts according to the manufacturer’s instructions.

#### 3.4.4. Neutral Lipid

Nile red staining is commonly used to measure intracellular neutral lipid content [66]. Algae cultures were properly diluted with sterile seawater. Then 2 µL of 0.1 mg/mL Nile red dye solution dissolved with acetone was added to the diluent with a volume of 200 µL, which was incubated at 25 °C for 10 min in the dark. Taking the algal solution without Nile red dye solution as a blank, the fluorescence intensity at 580 nm was detected with the excitation wavelength of 480 nm in a multi-mode microplate reader (Molecular Devices, SpectraMax M5/M5e, San Jose, CA, USA) [67]. The relative neutral lipid content was expressed as relative fluorescence units per algal cell (RFU/cell).

### 3.5. Photosynthetic Activity Analysis

The rapid fluorescence induction kinetics test (OJIP-test) was used to evaluate the photosynthetic performance of the microalgae. Photosystem II (PS II) maximum photochemical quantum yield *F_v_/F_m_*, the chlorophyll a fluorescence was measured by Os30p^+^ (Opti-sciences Inc., Hudson, NH, USA). The parameter *F_v/_F_m_* was calculated as follows according to Strassserf and Srivastava [68]: *F_v_*/*F*_m_ = (*F*_m_ − *F*_0_)/*F*_m,_ where *F_v_* represents the chlorophyll fluorescence change between the maximum fluorescence *F_m_* induced by the saturation pulse and the initial fluorescence *F*_0_.

### 3.6. Extraction and Purification of Intracellular Polysaccharides

The polysaccharides were extracted from microalgae cells by hot-water extraction and ethanol precipitation. Lyophilized algae cells were extracted twice with distilled water using a ratio of 1:40 (*w/v*) at 90 °C with stirring for 1 h. The extracts were collected by centrifugation at 3500× *g* and 4 °C for 30 min.

Solid trichloroacetic acid was added to the concentrated extracts to reach a final concentration of 8% (*w/w*) and left at 4 °C for 3 h to remove proteins. The supernatants were collected by centrifugation at 13,000 rpm for 20 min and precipitated 4 times with volumes of 95% ethanol at 4 °C for 12 h. The precipitate was recovered by centrifugation (8000× *g*, 5 min), washed twice with 95% ethanol, and then freeze-dried.

The obtained samples were named Iz-N and Iz-S, which were the polysaccharides from *I. zhangjiangensis* cultivated under nitrogen or sulfur deprivation, respectively, coupled with high light exposure.

### 3.7. Chrysolaminarin Content in the Extracted Polysaccharides

To determine the content of chrysolaminarin in the extracted polysaccharides, the enzyme kit “Mushroom and yeast beta-glucan assay procedure” (K-YBGL 02/21, Megazyme, Bray, Ireland) was used following the manufacturer’s instructions.

### 3.8. Fourier Transform Infrared Spectroscopy (FTIR)

The potassium bromide (KBr) tablet compression method was used for infrared spectroscopy analysis of the polysaccharide samples [69]. 1–2 mg of each polysaccharide sample was ground with dry spectroscopic grade KBr powder in an agate mortar, and was then pressed into thin slices with a tablet machine for infrared spectroscopy scanning with a wavenumber range of 4000–390 cm^−1^.

### 3.9. Antioxidant Activity Assessment

#### 3.9.1. Hydroxyl Radical Scavenging Activity Assay

The hydroxyl radical scavenging activity was assessed according to Yang et al. [69] with minor modifications. A 1 mL volume of polysaccharide solution at each different concentration, 6 mmol/L FeSO_4_ solution, and 6 mmol/L H_2_O_2_ were added into the test tube respectively, and incubated for 10 min. Then, 1 mL of 6 mmol/L salicylic acid was added. The absorbance was measured at 510 nm after 30 min.

Hydroxyl radical scavenging activity (%) = 1 − (A_1_ − A_2_)/A_0_ × 100%

where A_0_ represents the absorbance value of the blank group (replacing the sample with distilled water); A_1_ represents the absorbance value of a certain concentration of polysaccharide solution; A_2_ represents the absorbance value of polysaccharide solution without adding salicylic acid but with distilled water.

#### 3.9.2. DPPH Radical Scavenging Activity Assay

The scavenging activity of 1,1-Diphenyl-2-picrylhydrazyl (DPPH) radical was determined based on Xia et al. [19]. In short, 2 mL of 0.16 mmol/L DPPH solution (in 95% ethanol) was added to 2 mL of sample solutions of different concentrations, and placed at 37 °C for 30 min in the dark. Then the solutions were centrifuged at 5000 rpm for 5 min. The absorbance of the supernatant was measured at 517 nm. The DPPH radical scavenging activity was calculated as follows:

DPPH radical scavenging activity (%) = (B_0_ − B_1_)/B_0_ × 100%

where B_0_ represents the absorbance value of the blank group (replacing the sample with distilled water), and B_1_ represents the absorbance value of the sample.

#### 3.9.3. Ferric Reducing Antioxidant Power (FRAP) Activity Assay

The ferric reducing antioxidant power (FRAP) activity was determined based on Du et al. [57]. Briefly, the FRAP reagent contained 10 volumes of 300 mmol/L acetate buffer solution at pH 3.6, 1 volume of 10 mmol/L TPTZ with the solvent of 40 mmol/L HCl, and 1 volume of 20 mmol/L FeCl_3_·6H_2_O. A mixture containing 900 µL of freshly prepared FRAP reagent, 30 µL of polysaccharide sample solution and 90 µL of distilled water was incubated at 37 °C for 30 min, and the absorbance was measured at 593 nm. FRAP value was expressed as micromoles of Fe^2+^ equivalent per 1 g of sample using the calibration curve of Fe^2+^ made with FeSO_4_.

### 3.10. Statistical Analysis

Results were expressed as mean ± SD from three independent experiments. IBM SPSS Statistics 25.0 software was used to perform the statistical analysis. Multiple group comparisons were performed using one-way analysis of variance (ANOVA) and Fisher’s LSD. Values of *p* < 0.05 were defined as statistically significant.

## 4. Conclusions

The marine microalga *I. zhangjiangensis* was able to photoautotrophically accumulate large amounts of chrysolaminarin under nutrient deprivations with the order of -S > -N > -P, while high light exposure further improved chrysolaminarin production. -S enabled better chrysolaminarin production than -N by inducing a more suitable stress level and redirecting more carbon toward chrysolaminarin rather than neutral lipid accumulation. The chrysolaminarin produced from *I. zhangjiangensis* under HL-S and HL-N conditions had certain antioxidant activities, with Iz-S displaying an overall better activity, which was comparable to the widely used yeast β-glucan. *I. zhangjiangensis* could be a promising candidate for photosynthetic production of bioactive chrysolaminarin from CO_2_, with the possibility of regulating the activity via adjusting cultivation conditions.

## Figures and Tables

**Table 1:** Biomass productivity, chrysolaminarin productivity, chrysolaminarin content, neutral lipid content, and protein content of *Isochrysis zhangjiangensis* cultivated with different nutrient deprivations under low light (LL) and high light (HL) intensities. Values are expressed as mean ± standard deviation of three biological replicates. Values with different superscript letters in the same row represent significant differences (*p* < 0.05) between various cultivation conditions.

Culture Conditions	LL (Day 3)				HL (Day 4)			
	NR	-N	-P	-S	NR	-N	-P	-S
Biomass productivity (mg/L/day)	524.60 ± 80.67 ^a^	213.17 ± 15.57 ^c^	480.20 ± 10.89 ^a^	313.58± 7.36 ^b^	640.73± 22.86 ^a^	230.67± 12.67 ^d^	562.52 ± 25.44 ^b^	290.90 ± 10.82 ^c^
Chrysolaminarin productivity (mg/L/day)	15.23 ± 0.68 ^d^	90.31 ± 5.25 ^b^	41.34 ± 5.12 ^c^	145.93± 1.61 ^a^	55.06 ± 2.64 ^d^	98.71 ± 5.71 ^c^	132.39 ± 4.41 ^b^	155.08 ± 5.94 ^a^
Chrysolaminarin content (pg/cell)	3.48 ± 0.69 ^d^	40.97 ± 1.90 ^b^	8.95 ± 0.84 ^c^	45.31 ± 1.30 ^a^	8.71 ± 0.09 ^d^	40.58 ± 1.21 ^b^	23.10 ± 1.56 ^c^	50.72 ± 1.50 ^a^
Neutral lipid (RFU/cell)	22.86 ± 4.21 ^c^	109.30 ± 31.68 ^a^	32.37 ± 10.50 ^bc^	64.80 ± 6.25 ^b^	18.23 ± 8.67 ^d^	119.73 ± 3.60 ^a^	38.32 ± 4.37 ^c^	55.49 ± 7.28 ^b^
Protein (pg/cell)	31.19 ± 2.46 ^a^	19.83 ± 0.78 ^b^	26.80 ± 3.52 ^a^	18.38 ± 0.72 ^b^	22.30 ± 0.48 ^a^	16.65 ± 1.12 ^b^	17.19 ± 1.60 ^b^	15.20 ± 1.77 ^b^

**Table 2:** Comparison of different algal strains for chrysolaminarin production under photoautotrophic cultivation modes reported in the literature.

Strain	Culture Conditions		Biomass Productivity (mg/L/day)	Chrysolaminarin Content (%DW)	Chrysolaminarin Yield (mg/L)	Chrysolaminarin Productivity (mg/L/day)	Reference
*Isochrysis zhanjiangensis*	HL (150 μmol photons m^−2^ s^−1^)	-N	231	31.90	407	98.7	This study
		-S	291	41.71	632	155.1	
*Tribonema utriculosum*	Initial nitrogen concentration	3 mM	-	10.70	664	-	[49]
		9 mM	-	14.66	815	-	
		18 mM	-	14.17	757	-	
*Rhodosorus* sp. SCSIO-45730	Phosphate concentration	0 mg/L	114	6.6	191	8.3	[50]
		120 mg/L	541	19.4	2386	108.1	
		240 mg/L	582	14.5	1914	86.1	
*Odontella aurita*	Nitrogen concentration	6 mM	238	60.33	2383	142.7	[23]
		18 mM	373	46.27	2702	161.5	
*Phaeodactylum* *tricornutum*	Nitrogen concentration	14.5 mM	339	17.1	693	73.6	[22]
		2.9 mM	292	14.66	403	58.0	
*Odontella aurita*	LL (100 μmol photons m^−2^ s^−1^)	H-N (18 mM)	304	59.33	2397	240.0	[19]
		L-N (6 mM)	323	34.05	1440	144.0	
	HL (300 μmol photons m^−2^ s^−1^)	H-N (18 mM)	324	63.11	2676	268.0	
		L-N (6 mM)	536	48.16	3063	306.0	
*Odontella aurita*	LL (150 μmol photons m^−2^ s^−1^)	L-N (3.53 mM)	191	50.4	1824	117.7	[21]
		L-P (0.36 mM)	167	29.2	952	59.6	
		L-Si (0.11 mM)	290	43.1	2198	142.6	
		L-S (8.17 mM)	350	45.4	2724	177.7	
	HL (300 μmol photons m^−2^ s^−1^)	L-N (3.53 mM)	97	45.3	997	62.6	
		L-P (0.36 mM)	150	27.5	825	51.1	
		L-Si (0.11 mM)	337	42.3	2453	159.6	
		L-S (8.17 mM)	363	43.4	2691	175.5	

**Table 3:** Chemical composition of the extracted polysaccharides from *I. zhangjiangensis* cultivated under nitrogen or sulfur deprivation coupled with high light exposure. The extracted polysaccharides were named Iz-N and Iz-S, respectively. Values are expressed as mean ± standard deviation of three technical replicates.

Polysaccharide	Carbohydrate (%)	Chrysolaminarin (%)	Protein (%)	Lipid (%)
Iz-N	90.77 ± 0.57	86.55 ± 0.26	1.33 ± 0.083	0.09 ± 0.04
Iz-S	89.12 ± 1.03	87.07 ± 0.00	0.04 ± 0.003	0.41 ± 0.04

## Data Availability

All data are contained in the manuscript.

## Supplementary Materials

The following supporting information can be downloaded at: https://www.mdpi.com/article/10.3390/md20060351/s1, Figure S1: Cell morphology of Isochrysis zhangjiangensis cultivated with different nutrient deprivations (NR, nutrient repletion; -N, nitrogen deprivation; -P, phosphorus deprivation; -S, sulfur deprivation) under low light (LL) and high light (HL) intensities; Table S1: Composition of different nutrient-deficient medium.
