# Supplementary material for: Nutrient Deprivation Coupled with High Light Exposure for Bioactive Chrysolaminarin Production in the Marine Microalga Isochrysis zhangjiangensis
Source: Mar Drugs. 2022 May 26;20(6):351. doi: 10.3390/md20060351 (PMC9225646; doi:10.3390/md20060351)
Supplement: Supplementary file 1 [file marinedrugs-20-00351-s001.zip › marinedrugs-1717924-supplementary.pdf]

Table S1 Composition of different nutrient-deficient medium

Component	Final concentration in the growth medium			
	NR	-N	-P	-S
$NaNO_3$	600 mg/L	-	600 mg/L	600 mg/L
NaH_2PO_4	20 mg/L	20 mg/L	-	20 mg/L
Na_2SiO_3	30 mg/L	30 mg/L	30 mg/L	30 mg/L
$FeCl_3{\cdot}6H_2O$	3.15 mg/L	3.15 mg/L	3.15 mg/L	3.15 mg/L
EDTA·2Na·	4.36 mg/L	4.36 mg/L	4.36 mg/L	4.36 mg/L
$CuCl_2$	8.2 µg/L	8.2 µg/L	8.2 µg/L	8.2 µg/L
Na_2MoO_4	6.3 µg/L	6.3 µg/L	6.3 µg/L	6.3 µg/L
$ZnCl_2$	21 µg/L	21 µg/L	21 µg/L	21 µg/L
$CoCl_2{\cdot}6H_2O$	0.01 mg/L	0.01 mg/L	0.01 mg/L	0.01 mg/L
$MnCl_2{\cdot}4H_2O$	0.18 mg/L	0.18 mg/L	0.18 mg/L	0.18 mg/L
B_{12}	0.001 mg/L	0.001 mg/L	0.001 mg/L	0.001 mg/L
B_1	0.2 mg/L	0.2 mg/L	0.2 mg/L	0.2 mg/L
biotin	0.001 mg/L	0.001 mg/L	0.001 mg/L	0.001 mg/L
NaCl	21.22 g/L	21.22 g/L	21.22 g/L	24.02 g/L
$NaHCO_3$	0.174 g/L	0.174 g/L	0.174 g/L	0.174 g/L
$MgCl_2{\cdot}6H_2O$	9.034 g/L	9.034 g/L	9.034 g/L	9.034 g/L
$CaCl_2$	1.033 g/L	1.033 g/L	1.033 g/L	1.033 g/L
Na_2SO_4	3.407 g/L	3.407 g/L	3.407 g/L	-
KCl	0.357 g/L	0.357 g/L	0.357 g/L	0.357 g/L
KBr	0.0862 g/L	0.0862 g/L	0.0862 g/L	0.0862 g/L
H_3BO_3	0.023 g/L	0.023 g/L	0.023 g/L	0.023 g/L

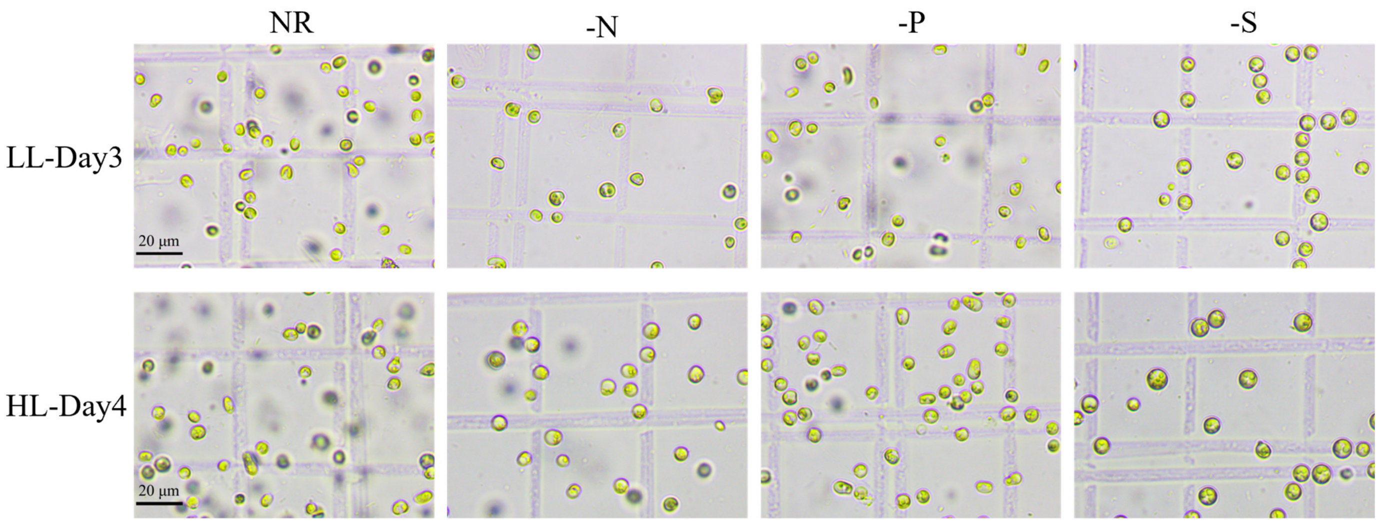

Figure S1. Cell morphology of *Isochrysis zhangjiangensis* cultivated with different nutrient deprivations (NR, nutrient repletion; -N, nitrogen deprivation; -P, phosphorus deprivation; -S, sulfur deprivation) under low light (LL) and high light (HL) intensities
